# Preconception Perceptions, Knowledge and Behaviours of Women With Gestational Diabetes Mellitus: A Qualitative Study

**DOI:** 10.1111/hex.70617

**Published:** 2026-02-26

**Authors:** Elana Payne, Danielle Schoenaker, Katrina Turner, Helen R. Murphy, Helen Skouteris, Khalida Ismail, Sergio A. Silverio, Madeleine Benton

**Affiliations:** ^1^ Department of Psychological Medicine, Institute of Psychiatry, Psychology and Neuroscience King's College London London UK; ^2^ Department of Women & Children's Health, School of Life Course & Population Sciences, Faculty of Life Sciences & Medicine King's College London London UK; ^3^ Department of Psychology, Institute of Population Health, Faculty of Health and Life Sciences University of Liverpool Liverpool UK; ^4^ School of Human Development and Health, Faculty of Medicine University of Southampton Southampton UK; ^5^ MRC Lifecourse Epidemiology Centre University of Southampton Southampton UK; ^6^ NIHR Southampton Biomedical Research Centre University Hospital Southampton NHS Foundation Trust, University of Southampton Southampton UK; ^7^ Population Health Sciences, Bristol Medical School University of Bristol Bristol UK; ^8^ Norwich Medical School University of East Anglia Norwich UK; ^9^ School of Public Health and Preventive Medicine Monash University Melbourne Australia; ^10^ Warwick Business School University of Warwick Coventry UK

**Keywords:** gestational diabetes mellitus, health behaviours, preconception, qualitative

## Abstract

**Background:**

Gestational diabetes mellitus (GDM) is one of the most common pregnancy complications. While considerable attention has been paid to the management of GDM during pregnancy, women's perceptions of GDM, knowledge of associated risk factors and health behaviours before and between pregnancies are less well understood, despite their importance for informing diabetes prevention programmes.

**Aims/Objectives:**

To explore women's knowledge of GDM risk factors, perceptions of GDM and preconception health behaviours.

**Methods:**

Individual semi‐structured interviews were undertaken with 23 women with GDM in the third trimester of pregnancy. Data were analysed using a template analysis based on preconception knowledge, risk communication, and perceptions and behaviours.

**Results:**

Women often had limited knowledge of GDM before pregnancy, with many first learning about the condition during antenatal screening. Perceptions of risk were largely weight or BMI centred, with less recognition of other factors. Although participants commonly described intentions to improve diet quality and increase physical activity prior to conception, these intentions were rarely translated into sustained behaviours. Reported barriers included time constraints, caregiving responsibilities, financial costs and limited access to clear, culturally appropriate guidance. Pregnancy preparedness varied by parity: women approaching a first pregnancy focused on general preparation and navigating the healthcare system, whereas women with a prior GDM experience planned around potential recurrence, including early self‐management and glucose monitoring.

**Conclusions:**

Findings support two priorities: (1) strengthening communication and education at the time of GDM screening and diagnosis so that results and next steps are clear, supportive and person‐centred; and (2) providing universal, general preconception support delivered proportionately to need, alongside targeted interconception pathways for women at higher absolute risk, following GDM. The findings do not imply universal GDM‐specific preconception education for all women; rather, they indicate a need for needs‐based communication during pregnancy and targeted interconception support delivered with clear signposting to resources.

**Patient or Public Contribution:**

An advisory group of seven women has been involved in this project. Four online sessions were conducted (between October 2023 and July 2024) to develop the research question, study materials, recruitment plans, interview schedules and participant retention plan.

## 1 Background

### 1.1 Gestational Diabetes Mellitus (GDM)

GDM is a common pregnancy complication. It affects around 14% of pregnancies globally, with rates continuing to rise [1]. GDM has important short‐ and long‐term physical and psychological consequences for mothers, babies and their families. It is associated with increased risk of pre‐eclampsia, caesarean delivery, preterm delivery, large for gestational age babies, and neonatal intensive care unit admission [2]. A range of socio‐demographic, obstetric and metabolic factors have been identified as contributing to the risk of GDM, including age, body mass index (BMI) and ethnicity [3, 4]. Current guidance within the UK recommends selective, risk‐based screening for women with one or more of the following risk factors: BMI ≥ 30 kg/m^2^; previous macrosomic infant (birthweight ≥ 4.5 kg); a first‐degree family history of diabetes; or minority ethnic background associated with a higher prevalence of diabetes. GDM is primarily managed through a combination of self‐monitoring of blood glucose, dietary and physical activity modification, with escalation to glucose‐lowering medication such as metformin or insulin, where required [5].

Although historically thought to commence in late pregnancy, emerging evidence suggests GDM may originate prior to conception and its effects often persist beyond pregnancy, driven by diverse metabolic pathways and B‐cell functionality [6, 7]. More recently, there have been calls to shift from a pregnancy‐centred approach to a broader, lifecourse perspective on GDM [6, 8].

### 1.2 Preconception Health

The preconception period is often framed as a window of opportunity for prevention and health promotion; however, engagement with preconception advice and capacity to act on it are heterogeneous and shaped by pregnancy intentions, social circumstances and access to support. Growing evidence highlights the preconception period as a critical window for supporting the overall health of non‐pregnant individuals of childbearing age (15–49 years) to improve pregnancy outcomes and long‐term health of current and future generations [9, 10]. The importance of preconception care is underpinned by the potential to improve suboptimal health behaviours and risk factors for pregnancy complications before a woman conceives [11]. It has been recognised as a period that offers opportunity for intervention, based on current thinking around foetal programming around the time of conception [12], lifecourse epidemiology and maternal motivation [10].

Weight, diet, physical activity and micronutrient deficiency are some of the modifiable factors examined in the preconception period for GDM risk [13, 14, 15, 16]. A recent meta‐analysis reported that women who gained weight before or between pregnancies were more likely to develop GDM than those whose weight remained stable [14]. Conversely, women who lost weight between pregnancies were less likely to develop GDM compared with women whose weight remained stable [14]. Micronutrient status before conception, particularly folate, has also been implicated in GDM risk [17]. Increasing research has suggested that low pre‐pregnancy vitamin B_12_ and high pre‐pregnancy vitamin B_9_ (folate) levels are associated with a higher risk of GDM [18]. For women with previous GDM, formal inter‑conception guidance remains limited; most international guidelines emphasise postpartum testing (e.g., an initial oral glucose tolerance test [OGTT] or haemoglobin A1C [HbA1c] at 6–13 weeks and annual HbA1c thereafter) and lifestyle advice to reduce risk of type 2 diabetes (T2D) [5]. This is despite the well‐recognised risk of GDM recurrence of around 50% in a subsequent pregnancy [19, 20].

An increasing number of interventions are being developed and tested to prevent GDM across the preconception, antenatal and inter‑conception periods. These have focused on diet, physical activity, supplementation and pharmacological intervention with evidence of varying effectiveness and reported challenges with reach and engagement [15, 21, 22, 23].

Within the United Kingdom, data specific to preconception perceptions, health knowledge, and behaviours in women with GDM is very limited [24] yet essential for effective development of interventions for the prevention of GDM, and to support informed pregnancy experiences, including engaging in proactive health behaviours before conception. Qualitative evidence from women with GDM indicates that limited pre‐diagnosis knowledge of GDM is associated with greater psychological distress and increased feelings of stigma following diagnosis [25]. International qualitative evidence mirrors these findings, with research showing that women across different settings often report limited knowledge of GDM prior to diagnosis, experience marked emotional distress at diagnosis and describe stigma characterised by guilt, shame and self‐blame [26, 27]. Addressing these preconception knowledge gaps is therefore critical to designing acceptable, well‐timed and engaging interventions, and to supporting informed pregnancy experiences through shared decision‐making, anticipation of care pathways and potentially reduced distress if GDM occurs [24, 28].

### 1.3 The Present Study

This study aims to address the knowledge gap regarding what women who develop GDM know, believe, and do before conception. Despite the well‐documented impact of factors such as diet, physical activity, micronutrient intake, age and family history on GDM risk, little is known about how women perceive and act upon these risks prior to pregnancy [29]. By exploring women's views and perceptions of GDM, this work will identify women's needs in the preconception period to inform future prevention strategies.

## 2 Methods

### 2.1 Study Design

This study was a component of a longitudinal qualitative study examining the impact of GDM on women's mental and physical health, service utilisation and barriers and facilitators to diabetes prevention programmes. The protocol for this study has been published [30]. The interview schedule (see Appendix S1) included questions regarding preconception perceptions of GDM, behaviours and knowledge, but it was not intended to be analysed separately. Ethics approval was granted by King's College London Research Ethics Committee (HR/DP‐24/25‐45503). All participants provided informed consent prior to interview participation (electronic consent for virtual interviews). Audio/video recording was undertaken with permission; transcripts were anonymised prior to analysis and stored securely in accordance with institutional data governance procedures. The study and analysis followed the Standards for Reporting Qualitative Research (SRQR) guidelines.

This study is situated within a post‐positivist research paradigm, underpinned philosophically by a critical realist ontology and an objectivist epistemology. The authors come from cross‐disciplinary professional backgrounds with expertise in women's health, psychology, diabetology, anthropology, epidemiology and psychiatry, who all have a shared interest in improving women's health. For full details on the study's philosophical underpinnings and positionality, please refer to the published protocol [30].

### 2.2 Recruitment and Data Collection

Women were recruited from across the UK via online pregnancy, women's health and GDM support communities (e.g., social media groups, forums and charities), using a maximum variation sampling approach [31], with specific focus on diversity in ethnicity, region and parity. Recruitment continued until maximum variation targets were met and the data set provided sufficient information power for the study aims, with no substantively new codes emerging within the three analytic domains. Additionally, our sampling strategy aimed to capture variation in prior GDM experience by including women who had experienced GDM once as well as those with multiple prior experiences. Recruitment finished when an adequate spread of demographics was achieved. Summary of participant characteristics can be found in Table 1.

**Table 1:** Summary of participant characteristics.

Characteristics	*N* (%)
Age	*M* = 32.4 ± 4.6 (range = 23–43)
Parity	
Nulliparous	15 (65.2%)
Multiparous	8 (34.8%)
Ethnicity	
White British	14 (60.9%)
South Asian—Bangladeshi	4 (17.4%)
South Asian—Indian	2 (8.7%)
South Asian—Pakistani	2 (8.7%)
Black African and White British	1 (4.3%)
Education	
University	18 (78.3%)
Sixth form/college	2 (8.7%)
School	1 (4.3%)
Apprenticeship	1 (4.3%)
Unknown	1 (4.3%)
Participant reported reason for being offered screening^a^	
BMI	10 (43.5%)
Ethnicity	8 (34.8%)
Family history	4 (17.4%)
Large for gestational age baby	3 (13%)
Previous GDM	2 (8.7%)

a Participants could select more than one reason for screening; totals therefore exceed *N* = 23. Reasons reflect participants' understanding of why screening was offered (as communicated by services), not extracted from clinical records.

Inclusion criteria are presented in Box 1 and full eligibility criteria (including exclusion criteria) are provided in the published protocol [30].

Box 1: **Inclusion Criteria**

- A self‐reported formal diagnosis of GDM more than two weeks prior to the first interview.
- More than 16 years of age
- Resident in the United Kingdom
- Can read and speak English, or a language in which an interpreter is available.

Women who had a GDM diagnosis (*N* = 23) were interviewed at around 35 weeks' gestation. This time point was selected to ensure women had sufficient time to experience GDM. Interviews were semi‐structured [32], conducted either virtually or in‐person [33] and lasted between 37 and 107 min (*Mean* = 59.6 min). All interviews were conducted by one researcher (MB), a chartered psychologist with expertise in qualitative methods and GDM research, and where required, an interpreter for women who did not speak English, following established protocols for sensitive research [34]. Interviews were audio and/or video recorded, transcribed verbatim, anonymised and imported into NVivo v.14 for data management and analysis. A £20 gift voucher was offered to participants after the interview in acknowledgement of their time and participation. Field notes were taken during the interview (M.B.) and upon familiarisation with the transcripts (EP). Reflections on the interview and any additional interactions were documented and recorded in a separate Microsoft Word document, while NVivo was used for data handling and analysis.

### 2.3 Analysis

The analytic template was derived from the interview questions specifically pertaining to preconception health (see interview schedule in Appendix S1). Adapting the interview questions as core components of the analytic template enabled the data to be organised initially into broad categories, which then supported more granular analysis and the development of sub‐themes. The purpose of this analysis was to understand women's experiences of preconception knowledge, risk communication and perceptions, and behaviours in the context of GDM; therefore, these three domains formed the overarching themes of our analytic template. Working with the data enabled the identification of sub‐themes to better capture the breadth and depth of participants' experiences. This approach is neither wholly deductive nor purely inductive, but abductive; that is, it is knowledge‐extending by drawing logical conclusions from the existing evidence (i.e., responses to specific questions) while also applying interpretive scrutiny to extend understanding in context [35].

To enhance rigour, we maintained an audit trail of analytic decisions (field notes, reflective memos and iterative versions of the coding template) and held regular analytic discussions within the multidisciplinary team to refine themes and resolve discrepancies.

In keeping with template analysis methodology [36], analysis commenced with reading the transcripts to ensure familiarity with the data set (Step 1; EP), alongside note‐taking to capture commonalities and differences in responses relating to preconception knowledge, risk factors, and behaviours. Preliminary codes were then generated (Step 2; EP). These preliminary codes were organised into an initial thematic template, which was iteratively developed and managed in NVivo (Step 3; EP). Data were initially coded at a broad level under the three overarching themes aligned to the interview questions, with working definitions applied to each theme (Step 4; EP). Following discussion with senior researchers (M.B. and S.A.S.), the data were coded in greater detail to refine the template and develop meaningful sub‐themes, and the revised template was then applied across the full data set (Step 5; EP). The final template comprised: ‘Preconception Perceptions of GDM’ (sub‐themes: (1) *Unexpected Discovery*, (2) *Familiar Foundations* and (3) *Diabetes by Association*); ‘Preconception Knowledge of GDM Risk Factors’ (sub‐themes: (1) *BMI‐Centric Risk Lens* and (2) *Fragmentary Awareness*); and ‘Preconception Health Behaviours’ (sub‐themes: (1) *Health Behaviour Intentions versus Implementation*, (2) *Weight Over Wellness* and (3) *Preconception Unpreparedness*). Final theme and sub‐theme names were agreed through team discussion and consensus (Step 6; EP,MB and SAS).

## 3 Results

Three main themes are reported from the template analysis: ‘Preconception Perceptions of GDM’, ‘Preconception Knowledge of GDM Risk Factors’ and ‘Preconception Health Behaviours’. Each theme has a number of sub‐themes, and illustrative quotations have been selected and presented in the thematic tables below.

### 3.1 Theme 1. Preconception Perceptions of GDM

Perceptions of GDM varied widely and reflected differences in participants' prior exposure to the condition. Some women were familiar with GDM because they had experienced the condition in a previous pregnancy. Others had heard of GDM through friends or family members, which gave them awareness of the condition but limited understanding of its management. Some participants had general knowledge of diabetes, most commonly T2D, which shaped their initial interpretation of GDM. However, most women reported that they were not familiar with GDM before being booked for screening. This theme comprises three sub‐themes: (1) *Unexpected Discovery*, (2) *Familiar Foundations* and (3) *Diabetes by Association*. See Table 2 for relevant quotations. It is important to note that providing data to one of these sub‐themes did not preclude women from providing data to one of the other sub‐themes (e.g., some women had familiarity with diabetes through family members, while also having had previous experience with GDM).

The *Unexpected Discovery* sub‐theme describes women who encountered GDM for the first time at screening and reported little or no prior awareness of the condition. Participants did not describe screening as a process they could engage with actively; instead, it was often experienced as a routine step in antenatal care rather than a decision point. This was largely because many women were unaware of GDM prior to screening, limiting their opportunity to ask questions about the rationale for screening, the procedures involved and the implications of the results. For these women, the diagnosis represented an abrupt change to their pregnancy experience and required immediate dietary and lifestyle adjustments. Information was often delivered in a single phone call, during which participants were expected to absorb a new diagnosis, decipher unfamiliar medical terminology and accept the sudden requirement for glucose monitoring, dietary restrictions and additional appointments with multiple healthcare professionals. Many described the experience as overwhelming, as they tried to reconcile fears for their baby's well‐being with the practical demands of monitoring their glucose levels, completing food diaries, and making decisions about medication—often without prior knowledge of GDM earlier in pregnancy. Participants in this sub‐theme also described feeling unprepared to anticipate or plan preconception health behaviours relevant to GDM risk.

*Familiar Foundations* highlights participants who drew upon insights from previous pregnancies or knowledge shared by friends and family to inform their initial understanding of GDM. Women with prior experience of GDM described familiarity with aspects of screening and management, such as glucose monitoring. Some participants anticipated GDM recurrence and reported feelings of anxiety or distress regarding potential impacts on their care, including birth options. In addition, women who had familiar foundations were more likely to view GDM through a *BMI‐Centric Risk Lens* and would perform health behaviours in line with *Weight Over Wellness*.

*Diabetes by Association* describes women who lacked specific knowledge of GDM but had some general awareness of diabetes, predominantly T2D, through family members or a healthcare background. Participants described drawing on this existing diabetes knowledge as a starting point for making sense of GDM once diagnosed, sometimes initially assuming that GDM would follow a course similar to T2D, and at other times emphasising the need to revise their understanding and recognise GDM as distinct. In this context, participants reported concerns about longer‐term implications, including worry about whether diabetes might persist after birth and increased risk of developing T2D later in life. Participants also described stigma and blame‐oriented assumptions and experiences of judgement, including suggestions from others and/or themselves that GDM was caused only by sugar intake or personal behaviours, alongside reports of self‐blame and of having done something wrong.

**Table 2:** Preconception perceptions of GDM.

Unexpected Discovery	“But certainly, pre‐conception, I had no idea of any of the ins and outs and what it entailed.” P11
	“I knew absolutely nothing about it.” P19
	“Um not much, really. Nothing really. Nothing at all.” P10
	“I don't think it was ever given as a choice. It was just, So we will send you for a GTT at this gestation.” P18
	“…other than ‘I will need to take a test, it will mean drinking something incredibly sugary’ I didn't kind of know anything beyond that … what it meant.” P2
	“Before that … I didn't hear about gestational diabetes. … I came here in the UK … I searched for it on Google and learned from there.” P16
	“I'd heard the term gestational diabetes … but that was the end of my knowledge of it.” P20
	“I went for one booking appointment and then I got given like fifty million appointments after that. And so I just went for all of them, I didn't really question it, just went for all of them, and one of them was the glucose test.” P6
	“I didn't realise at the time but it was actually the GDM clinic. So, without telling me I was at increased risk of it, I was kind of just booked in for it and then around that time I then also got a letter from the diabetes team, the midwifery diabetes team, um to go and collect a kit. And again it wasn't communicated to me, these letters just turned up. I was just kind of booked into it?” P15
Familiar Foundations	“I had gestational diabetes back in twenty‐nineteen in one of my other pregnancies … This pregnancy, I've kind of relied on the knowledge I've gained previously.” P4
	“To be perfectly honest, I probably would have declined the growth scans if I didn't know what a fight it was gonna be…I probably shouldn't have, but I've kind of just accepted them because I can't deal with the coercion and the fight this time round…So I‐ I then went and read every article, every scientific paper I could on gestational diabetes, on the management of it, on outcomes, on…mode of birth… Literally read everything that I could find on it.” P18
	“…so this time I've been quite anxious to try and get to thirty‐seven weeks so that I could…have a home birth originally and then all their faces change when you mention home birth. I know cos they're not overly keen on it.” P4
	“So I knew about it because my sister had it in her pregnancy and my sister‐in‐law had it.” P5
	“So I had it with my son … I was very, very oblivious when I got diagnosed the first time around.” P23
	“I'm a nurse so obviously had that nurse background … I was aware of it already and I was worried that I would get it.” P1
	“It was more, ‘Oh, God, it's going to ruin my chances of the birth that I want’.” P18
	“My only kind of awareness of it was vaguely that a family friend's niece had it, and then I didn't really know anything about it myself until they put me down for testing due to my BMI at booking.” P22
Diabetes by Association	“I'm quite familiar with diabetes in general purely because pretty much everyone in my family has type 2 … I've grown up with diabetes. I wasn't very familiar with gestational diabetes.” P18
	“…people… were like ‘Oh is it because you ate loads of sugar’….” P17
	“I assumed it kind of pretty much followed type 2 diabetes … so I thought when I got it … that maybe it was something I had done.” P23
	“But I didn't know a lot about gestational diabetes at that point… I hadn't come across anything to do with gestational diabetes beyond it being something you'd read quickly about in a book.” P11
	“I'm a nurse anyway so I knew about diabetes regardless… gestational diabetes is its own sort of concept… It was a lot about relearning… and building on what I already knew.” P6
	“They make me feel like I am doing something wrong that's why I diagnosis GDM.” P14
	“I don't want to be stuck with this after I've had the baby.” P10
	“Um ((Sighs)) I knew a little bit about … diabetes, my mum and my father‐in‐law both had it … but I didn't realise that gestational diabetes was the thing.” P15

### 3.2 Theme 2. Preconception Knowledge of GDM Risk Factors

Knowledge of GDM Risk Factors was generally limited and often rudimentary. Most women entered pregnancy with only limited knowledge, if any, of the factors which increased risk, often learning risk factors only when screening was discussed. Analysis generated two sub‐themes which captured this spectrum of understanding: (1) *BMI‐Centric Risk Lens and* (2) *Fragmentary Awareness*. See Table 3 for relevant quotations.

The *BMI‐Centric Risk Lens* sub‐theme describes women who perceived their risk of GDM as being primarily attributable to their body size. This perception often stemmed from interactions with healthcare professionals, in which weight and BMI were frequently emphasised in discussions about preconception and antenatal health. During healthcare encounters, midwives commonly identified elevated BMI as a risk factor for various complications, fertility clinics imposed strict BMI thresholds prior to IVF treatment, and OGTT screening was often justified largely on the basis of weight. Discussions of GDM risk rarely extended beyond BMI. Importantly, several women who self‐reported carrying extra weight described entering pregnancy anticipating stigma related to their weight. They reported being accustomed to narratives in which weight was positioned as the underlying cause of multiple health conditions and, consequently, anticipated pregnancy complications more broadly, an expectation that persisted into their GDM care.

*Fragmentary Awareness* captures women who arrived at conception with little to no understanding of GDM risk factors. Several stated explicitly that they did not know how GDM develops or why individuals are diagnosed, while others described only vague awareness, such as ‘running in the family’, without being able to identify risk factors in detail or recognise their relevance to themselves. Linguistic barriers and a lack of culturally relevant information further limited understanding among participants whose first language was not English, prompting reliance on informal sources or post‐diagnosis internet searches. Many women described perceiving themselves to be at low risk because they did not consider themselves to have the typical risk factors and reported feeling shocked or surprised when diagnosed following screening. For these women, screening and diagnosis represented the first clear point at which their personal risk became apparent.

**Table 3:** Preconception Knowledge of GDM Risk Factors.

BMI‐Centric Risk Lens	“… so I had no knowledge of the ins and outs of it. The midwife in the early appointments had mentioned it. Kind of just said that my high BMI was gonna be the primary risk factor. Um, so that's why I would be put forward for a test.” P11
	“I'll be honest, I was quite naïve … I knew that being over a certain BMI was a risk factor, but I didn't think I had any other high‐risk factors.” P17
	“We had some issues with our midwife and quite a lot of it was based on my BMI. Like, my whole pregnancy has been classified as higher risk automatically because of my BMI.” P5
	“No, I just got put forward, because my BMI was just over.” P19
	“So, I don't think that I'd considered that for GDM. I assumed because I'm larger that I'd have like higher risks of having anything. But it's [GDM] not spoken about the same way other things are spoken about. And so, this wasn't really on the radar, and it wasn't something that I was conscious of.” P15
	“I think the reason why I've been referred for it is because my BMI is point one over, I think, which is like, the threshold.” P6
	“When I did go private, they had a strict BMI limit, and it was like, if my BMI was over a certain point, I'd have to weigh in a couple of weeks before each procedure [IVF], and if my BMI was over, they'd cancel the round. So um, they were quite strict.” P15
	“I didn't have any symptoms at all. I just got sent for that sugar test because of my BMI.” P22
Fragmentary Awareness	“Nothing. Like literally knew nothing about it. I have heard the term gestational diabetes before, but I didn't know what it meant. Um I didn't know the testing, the screening, anything. I know friends had said that it was a nasty glucose drink. That was kind of the end of my knowledge of it.” P20
	“I didn't really know any of the science; I didn't really know how people got it or why people were diagnosed with it.” P12
	“They test Asians earlier because of hereditary but nothing more than that.” P21
	“I wasn't aware of them [risk factors] I know I got tested ‘cause my mum had it and obviously my ethnic background as well. But again, I found that out in the pregnancy.” P23
	“This is my first pregnancy, so I didn't know the risk factors, the only thing I know is you have gestational diabetes if you are overweight or you have it in your family. That's all I knew.” P3
	“Um, I think everything I knew about gestational diabetes prior to being diagnosed came from social media. So I was quite anxious just knowing a lot of people have it but again it was kind of a negative portrayal of it. But it was all based on like the algorithm and like searching. And then I went down quite a bit of a rabbit hole.” P12
	“Yeah, I didn't really know about specific factors, as far as I'm aware, it's like a family history thing as well um or being overweight but beyond that, I didn't really know about specific factors for developing gestational diabetes because I don't really have any specific like risk factors. In fact, I'm‐ when I went for my glucose test, they were a bit surprised that I'd been sent for a glucose test cos I didn't think I meet the criteria to have gone for one.” P6
	“It was shocking for me to have GDM as I was not expecting to have it. I'm not overweight and very picky about my foods.” P14
	“I knew about it running in the family but in terms of other risk factors, no, I don't recall knowing any of the risk factors.” P12

### 3.3 Theme 3. Preconception Health Behaviours

Preconception health behaviours included dietary changes, physical activity and weight loss. In discussing women's approaches to preconception health behaviours, three sub‐themes were identified**:** (1) *Health Behaviour Intentions Versus Implementation*, (2) *Weight Over Wellness* and (3) *Preconception Unpreparedness*. See Table 4 for illustrative quotations.

The *Health Behaviour Intentions Versus Implementation* sub‐theme encompasses women who described intending to optimise their health prior to conception, with a focus on diet, weight and physical activity. While participants commonly expressed the importance of preconception health, many described these intentions in general terms and reported making few specific or sustained changes when trying to conceive. Some women described modest, feasible adjustments, such as reducing portion sizes, cutting down on carbohydrates, or engaging in varied forms of physical activity (e.g., Pilates or exercise classes), and several reported taking supplements in the preconception period. However, participants described considerable variability in how consistently these behaviours were sustained over time, with healthier eating or exercise routines often difficult to sustain, particularly following competing life events. Most health behaviour changes were self‐directed, with women relying on personal judgement rather than professional guidance. When asked to describe their behaviours in detail, many participants spoke broadly about having improved their lifestyle, without specifying the nature, intensity, or duration of changes. Healthier behaviours were often assumed to be beneficial for the baby, with less explicit consideration of maternal health outcomes or women's health more broadly.

*Weight Over Wellness* described participants who placed a strong emphasis on weight or BMI before pregnancy, often overshadowing broader, holistic and long‐term health practices. Several women described actively attempting to lose weight before conception, including substantial weight loss and periods of dieting or increased exercise. Preconception preparations were further shaped by fertility treatment requirements, in which achieving a specified BMI was positioned as a condition of eligibility. However, the rationale for these thresholds was not consistently explained. Practical, evidence‐based strategies for achieving healthy weight reduction were seldom provided, leaving women feeling confused and frustrated. In one account, the emphasis on reaching a specific numerical target contributed to a short‐term crash diet prior to treatment and was described as shifting focus toward the number on the scales rather than broader health behaviours. Participants also reflected on the psychosocial complexity of pursuing weight loss, including tensions with fat‐positive and body‐positive values. Several participants explicitly described rapid weight loss as unsustainable and associated it with weight regain over time.

*Preconception Unpreparedness* was the final sub‐theme. Some participants described undertaking little or no active preparation before conception. For some, pregnancy occurred unexpectedly, and they reported making no preparatory changes. Others described intending to be healthy but not implementing specific behaviours, or finding that healthier routines were difficult to sustain alongside major life events or day‐to‐day demands. Several participants described minimal engagement with preconception physical activity. A small number of participants also described communication barriers when receiving information, including the reliance on interpreters and limited confidence in English. These barriers prompted some women to seek information independently online to help them understand health guidance before pregnancy.

**Table 4:** Preconception health behaviours.

Health Behaviour Intentions Versus Implementation	“Food and exercise I was more conscious of. Not folate.” P21
	“I always maintained like a regular exercise regime where I do at least two exercise classes a week, and I was taking, um, pre pregnancy vitamins. Diet wise I was mostly being quite controlled.” P13
	“I'd lost about five stone since I last got pregnant and so I tried to focus on getting healthier and losing weight and exercising more before conception. Um and I was taking folic acid and multi vits.” P18
	“When we were trying for a baby, it was always that ‘right well, you're anaemic, so you need to make sure that you keep taking these folic tablets three months before you conceive’. I'm quite active anyway in terms of I play football or I played football before I got pregnant, three times a week, so it a‐ it was always keeping fit for me.” P20
	“Yes, I was taking the prenatal vitamins and stuff, but I don't think I was particularly good with my diet.” P23
	“I like to think I was doing the right kind of things, in terms of when I was trying to conceive, but I hadn't really done anything specifically out of my way.” P6
	“I mean, in the run up to like that conception, I definitely was really mindful about what I was eating, and exercise…, because it was in the run up to the wedding, it was the healthiest I think I've ever been. And then after the wedding everything slipped and I didn't go to the gym for months after. So, no, I wasn't particularly mindful about what I was eating while we were trying. It was more in the run up to the wedding.” P22
	“I would say I dipped in and out of dieting. I was definitely more conscious of what I was eating, because in the run up to, conception, I was trying to lose weight. I had taken some weight off, by cutting down on carbs and also I did take folic acid and some other vitamins.” P19
	“we kind of… try and be a little bit more health conscious and more exercise and I started doing an online subscription to a dance class which was three times a week dance class and I was just eating less, like, not having a whole pan of macaroni and cheese without batting an eyelid.” P4
	“I think, again, probably weight and diet and exercise, um I did a lot of pilates, and I was definitely very mindful of what I was eating, and I did take pre pregnancy vitamins.” P17
Weight Over Wellness	“I was really keen to try and get into the healthiest place before we actually conceived. I managed to lose, eleven stone before we got pregnant.” P11
	“…so from my 2021 pregnancy to this pregnancy, I was quite focused on trying to lose weight.” P4
	“I had the surgery [gastric sleeve] and then I was trying to, get my BMI down further to be eligible for treatment on the NHS, and I was eligible for some treatment, but not full IVF. And then we ended up going to this private fertility clinic and they had a really strict BMI limit.” P15
	“And it is kind of hard when you're trying to sort of focus on weight loss without going against the culture of fatphobia in our society, because I'm trying to be quite fat positive and body positive.” P18
	“I've always, I dip in and out of dieting, but carbs, especially for me, is a sort of…in order for me to lose any weight, along with exercise, it's carb related.” P19
	“I did lose something like three stone in a matter of, like‐ like, a couple of months before, but also it's not sustainable either, which is why I put it all back on, not all of it, but most of it.” P22
	“Yeah, so I'd lost about five stone in the two‐three years before getting pregnant … I'd been focused on losing weight, well, more about feeling healthier, but as a consequence … it was losing weight.” P18
Preconception Unpreparedness	“God … no, this baby was a bit of a surprise, so there wasn't really anything [I did].” P7
	“Um no, not really. Nothing, nothing particular.” P10
	“Before pregnancy … no, I didn't do any exercise.” P16
	“I wasn't particularly mindful when we were, trying. … I'd stopped going to the gym, stopped being as healthy as I should.” P22
	“No, nothing at all, and I don't know if it's a taboo subject, or it's they don't teach you it in school, I can tell you that they definitely didn't teach us about it in school and when you're trying to get pregnant, they don't necessarily talk about it a lot then either so I didn't know about it.” P20
	“Um, losing weight had been quite difficult just due to the fatigue issues that I was having with long COVID, so I had put more weight on over the last few years. Um and a lot of the doctors I've spoken to had talked to me about high risk, like preeclampsia, and all of this other stuff. So, I was more focused on the things that they were saying that I could be high risk for but gestational diabetes was never one of them. … Um, so yeah, it wasn't ever on my radar to try and think about how I might tackle that. ((laughs))” P8
	“…before I was living in Bangladesh, I didn't hear about gestational diabetes. But after coming here and I am affected by diabetes, I search for it in Google and learn from there about this thing. Before that I didn't hear this.” P16
	“I like to think I was doing the right kind of things … but I hadn't really done anything specifically out of my way.” P6

## 4 Discussion

### 4.1 Summary of Key Findings

This study explored preconception perceptions, knowledge, and behaviours among women with GDM, identifying gaps in understanding and opportunities for targeted support to inform future prevention strategies. Three overarching themes are presented: (1) ‘Preconception Perceptions of GDM’, (2) Preconception Knowledge of Risk Factors and (3) ‘Preconception Health Behaviours’. Together, these themes highlight variability in women's awareness of GDM prior to pregnancy, the prominence of weight‐centred framing of risk and challenges translating general health intentions into sustained health behaviours before pregnancy and beyond.

Many women entered pregnancy with limited awareness of GDM and described encountering the condition for the first time at screening. For these women, diagnosis represented an unexpected disruption to their pregnancy experience and necessitated rapid engagement with unfamiliar information and care processes. Perceptions of GDM risk were frequently framed around body weight and BMI, with comparatively little reference to other contributing factors such as age, family history, ethnicity or prior GDM. With regard to preconception health behaviours, participants commonly expressed broad intentions to ‘be healthy’ prior to pregnancy, but reported difficulties translating these intentions into specific, sustained actions. Barriers included competing life demands, limited time and uncertainty about the relevance and timing of specific behaviours. Experiences also differed by parity. Women planning a first pregnancy tended to seek general guidance on preparing for pregnancy and navigating healthcare services, whereas women planning a subsequent pregnancy, especially those who had experienced a previous GDM pregnancy, focused on recurring risk, glucose monitoring and interconception support.

### 4.2 Interpretation in the Context of the Literature

Limited awareness of GDM prior to pregnancy is well documented, and the findings of this study are consistent with existing literature indicating GDM is often first considered at the point of screening or diagnosis [24]. In line with previous qualitative work, some participants drew on general knowledge of diabetes, most commonly T2D, to interpret their diagnosis, which shaped early perceptions and expectations of GDM management [37]. In this study, the *Unexpected Discovery* of GDM underscored the challenges of risk communication at screening, particularly when women have limited preconception knowledge of the condition. Participants' accounts point to the importance of sensitive, staged communication that clarifies steps following diagnosis. This aligns with NICE guidance on person‐centred antenatal and diabetes in pregnancy care [5]. It is also important to recognise that although shared decision‐making is the preferred model in maternity care [38], the unexpected diagnosis of GDM can initially limit women's ability to participate fully until information has been provided and understanding has been developed.

Diabetes and weight are often treated synonymously in public and clinical discourse; however, GDM risk is driven by multiple interacting factors. Beyond adiposity, GDM risk is influenced by age, prior GDM, family history, ethnicity [39] and conditions such as polycystic ovary syndrome (PCOS) [4, 40]. Participants' accounts suggest that clinical explanations of GDM risk frequently foregrounded body weight or BMI, with limited discussion of other contributing factors, highlighting how risk communication may inadvertently prioritise weight‐based narratives.

Where explanations of the underlying pathophysiology were limited, including how nutrition, physical activity, placental function and insulin resistance contribute to GDM risk, women often interpreted BMI as both the dominant indicator of risk and the only modifiable factor. This pattern is consistent with qualitative research demonstrating that preconception knowledge often clusters around a narrow set of salient behaviours, rather than a broader, multifactorial understanding of risk [41, 42].

Participants' accounts also reflected the influence of wider societal and healthcare narratives that moralise women's bodies during the preconception, pregnancy and postpartum periods [43, 44, 45]. Weight stigma is well documented in maternity care and has been associated with reduced trust in healthcare, psychological distress and poorer engagement with health‐promoting behaviours [44, 45]. Rather than motivating behaviour change, weight stigma has been shown to undermine metabolic health through stress pathways and maladaptive coping behaviours, including reduced physical activity and disordered eating [44].

In pregnancy populations, experiences of weight‐related stigma have also been associated with gestational weight gain above recommendations and increased risk of postpartum depression, independent of baseline BMI [45]. In this context, the BMI‐centric framings identified in this study may inadvertently amplify self‐blame and distress at the point where women require clear, compassionate, and actionable support to navigate screening, diagnosis and care.

Although this study did not set out to assess the effectiveness of women's health behaviours in the preconception period, the findings highlight the complexity of behaviour change in the preconception period [4, 40]. Participants commonly acknowledged the importance of optimising health prior to pregnancy but described difficulties implementing and sustaining specific behaviours. These challenges were shaped by competing demands, limited access to tailored guidance and uncertainty about what changes were most relevant.

While the preconception period is frequently framed as a window of opportunity for intervention, these findings suggest that its utility is highly variable and contingent on pregnancy intention, life circumstances and access to support. For some participants, particularly those with unplanned pregnancies, there was no distinct preparatory phase prior to conception. Even among women actively planning pregnancy, competing responsibilities and limited guidance constrained their ability to implement sustained changes. This supports prior calls for the conceptualisation of preconception care as being beneficial for women's health across the lifecourse, rather than solely as a preparation for pregnancy [46, 47].

In light of these findings, it would appear beneficial to communicate nonmodifiable risks to women, and provide specific, feasible examples of diet and activity change, framed in a nonstigmatising, culturally appropriate and supportive way. This would align with previously published work surrounding preconception guidance and the wider consensus to avoid weight stigma in clinical communication [48, 49, 50]. It also aligns with women's desire for clinicians to reduce emphasis on weight alone, avoid fear‐based tactics and provide personalised advice that recognises individual circumstances and weight histories [45]. Participants' accounts also align with health psychology evidence describing an intention–behaviour gap, whereby intentions account for only a moderate proportion of subsequent behaviour in the absence of concrete planning and support [51, 52]. Consistent with this, women often described ‘healthy living’ in broad terms, with limited specification of the nature, intensity or duration of behavioural changes. Similar patterns have been reported in qualitative syntheses of preconception health behaviours [42].

For women with, or at risk of GDM, bridging this intention–behaviour gap is likely to require specific action planning alongside concise, credible and condition‐relevant guidance [53, 54]. Studies in GDM populations consistently show that women value clear, practical advice on diet and physical activity but encounter persistent barriers to implementing this advice in everyday life [54, 55].

### 4.3 Implications for Policy and Practice

The findings of this study suggest there are critical gaps between existing preconception guidance and women's experiences of support and implementation. First, the time of GDM screening represents an opportunity for knowledge mobilisation. Clear, plain language explanations of the purpose of screening, interpretation of results and immediate implications for care, paired with signposting to reliable resources, may help mitigate the disorientation described at diagnosis and support informed engagement with care pathways [5]. Second, rather than advocating universal GDM‐specific preconception education, our findings support the provision of universal, general preconception support that emphasises behaviours with broad benefits for both mother and infant across the lifecourse. These include dietary quality, folate supplementation, physical activity, smoking cessation, delivered opportunistically through primary care, contraceptive reviews, pharmacies, community settings and digital platforms [11, 56, 57], and delivery should be proportionate to need to address existing health inequalities [57, 58]. Third, our findings indicate that parity and prior experience shape women's informational needs. Women considering a first pregnancy may benefit most from building general health literacy about pregnancy preparation and navigating services, whereas women planning a subsequent pregnancy, particularly after GDM, may benefit from targeted interconception pathways which build on prior experience, addressing recurrence risk, lifestyle support, consistent with NICE guidance [59].

Across all groups, communication should avoid attributing risk to body size alone and instead present a clear, multifactorial model of GDM risk alongside personalised, actionable options for risk reduction and post‐diagnosis health. Finally, our findings and the wider literature suggest that care that considers weight stigma reduction should be considered a core component of both preconception support and GDM‐related care pathways, including the use of respectful, non‐judgemental language and avoidance of fear‐based messaging. Emerging implementation work suggests that co‐designed, stigma‐aware resources may be feasible and acceptable in antenatal settings and support more effective clinician–patient communication.

### 4.4 Strengths, Limitations and Future Research

A strength of this study is the timing of data collection during pregnancy and after participants had been diagnosed with GDM, which enabled participants to reflect on their preconception knowledge and behaviours in light of subsequent experiences. Our use of template analysis also supported systematic comparison of sub‐themes across participants. However, retrospective recall may have been influenced by post‐diagnosis learning and experiences. It is also important to acknowledge that the sample was, on the whole, well‐educated and in professional jobs, which may under‐represent lower levels of health literacy among more socially complex populations. Further research using prospective qualitative or mixed‐methods designs is needed to examine how knowledge, risk perceptions and behaviours evolve from preconception through early pregnancy, and to evaluate proportionate universal interventions by socio‐demographic factors such as deprivation and ethnicity gradients evident in current literature [57].

## 5 Conclusion

Women in this study varied widely in their preconception awareness of GDM. Many described encountering (and subsequently internalising) a weight‐centred framing of GDM risk, particularly where BMI was emphasised in clinical encounters; these views are therefore likely shaped by the advice and messaging women receive from healthcare professionals as well as wider societal norms about body weight. Women also described difficulties translating general intentions to ‘be healthy’ into specific, sustained actions, with patterns differing by parity and prior experiences. Taken together with existing literature, the findings support two potential priorities: (1) strengthening communication at the time of GDM screening to ensure clarity and support; and (2) delivering universal preconception support with proportionate, targeted interconception pathways for women at higher absolute risk, particularly following GDM. The findings do not go so far as to imply that all women should receive GDM‐specific preconception education; rather, they support a needs‐based communication during pregnancy and targeted stigma‐aware interconception support delivered without blame and with clear signposting to resources.

## Author Contributions

**Elana Payne:** conceptualisation, data curation, formal analysis, methodology, project administration, writing – original draft. **Danielle Schoenaker:** conceptualisation, writing – review and editing. **Katrina Turner:** conceptualisation, methodology, writing – review and editing. **Helen R. Murphy:** conceptualisation, methodology, writing – review and editing. **Helen Skouteris:** conceptualisation, writing – review and editing. **Khalida Ismail:** conceptualisation, writing – review and editing. **Sergio A. Silverio:** conceptualisation, methodology, validation, writing – review and editing. **Madeleine Benton:** conceptualisation, investigation, funding acquisition, methodology, data curation, writing – review and editing, supervision, project administration, resources.

## Ethics Statement

Ethical approval was granted on 5 November 2024 by King's College London Research Ethics Committee (HR/DP‐24/25‐45503).

## Conflicts of Interest

The authors declare no conflicts of interest.

## Supporting information

Appendix S1.

## Data Availability

The data for this study will be made available from the corresponding author upon reasonable request.
