# Supplementary material for: Preconception Perceptions, Knowledge and Behaviours of Women With Gestational Diabetes Mellitus: A Qualitative Study
Source: Health Expect. 2026 Feb 26;29(2):e70617. doi: 10.1111/hex.70617 (PMC12936987; doi:10.1111/hex.70617)
Supplement: Supplementary file 1 — Appendix S1. [file HEX-29-e70617-s001.docx]

**Interview schedule: Time point 1 (35 weeks gestation)**

**1. Pre-conception and Pregnancy Knowledge**

**GDM Knowledge:**

- Can you share what you knew about gestational diabetes, if anything, before your diagnosis?
- Were you aware of any risk factors for gestational diabetes prior to becoming pregnant?
- Were you aware you had been referred to the Gestational Diabetes service?

**Pre-conception Health and Behaviors:**

- Before becoming pregnant, did you have any health behaviours you were focusing on? (e.g., weight, diet, exercise) And what were you motivations behind them?

**2. Pregnancy Experience and Management

Information Provision:**

- Can you tell me about your experience of diagnosis? How was gestational diabetes explained to you?
- What kind of information did you receive? Did you feel it was sufficient?

**Interactions with Health Services:**

- Can you describe your experience with healthcare services in managing your gestational diabetes?
- Were there any particular healthcare professionals who made a significant impact on your care?

**Management Experience:**

- How did you manage your gestational diabetes during pregnancy (e.g., diet, exercise, medication)?
- How did management impact on your experience of pregnancy?
- Were there any challenges you faced in managing it?

**Psychological Impact:**

- Can you describe how being diagnosed with gestational diabetes has affected you emotionally?
- Did you experience any particular fears, anxieties, or stress during your pregnancy because of gestational diabetes?
- How do you think your diagnosis of gestational diabetes impacted how you felt about your pregnancy?
- How do you think your diagnosis of gestational diabetes impacted how you felt towards your baby whilst pregnant?
- Did a diagnosis of GDM impact your eating behaviours or thoughts around food in any way?
- What was your biggest concern with being diagnosed with GDM for you and your baby?

**Partner and Family Involvement:**

- How involved were your partner or family members in your experience of gestational diabetes?
- Did they provide support? If so, how?

**Stigma**

- Do you feel you have been treated differently by other people because of the gestational diabetes?

**3. Labour and Birth**

**Information Provision:**

- What information have you received regarding the potential impact of gestational diabetes on labour and birth?
- Has the diagnosis impacted on how you have thought about types of birth?

**4. Breastfeeding**

**Intentions and Perceived Barriers:**

- Have you thought about how you would like to feed your baby after birth, for example breast or formula feeding?
- Why have you decided to feed your baby this way?

**Information Received:**

- What information have you been given about breastfeeding, particularly as it relates to having had gestational diabetes?
- Have you sought any additional information yourself?

**Understanding Impact/Benefits for Mother and Infant:**

- How do you understand the impact of breastfeeding for both you and your baby after a gestational diabetes diagnosis? (e.g. benefits of energy expenditure when breast feeding (circa 500 Kcals a day))
- Did your healthcare providers explain any specific impacts?

**5. Postnatal Knowledge**

**Immediately After Birth:**

- Have you received any advice on what to do after birth? (e.g. 12-weeks testing)
- What information have you received about the impact of GDM straight after birth?
- Do you know how baby is monitored immediately after they are born in terms of GDM?
- Do you know how you need to monitor yourself immediately after baby is born?

**General Knowledge**

- Are there any concerns you have around how gestational diabetes might impact you in the postnatal period? (your health or babies health)
- Are there health behaviours that you are hoping to continue or change in the postnatal period?
- What would support you in making these changes?
- Has the way you think about your health in general changed since you were diagnosed with GDM?
- What have you been told about your risk of developing type 2 diabetes after your pregnancy?

**Closing:**

- Is there anything else you would like to share about your experience with gestational diabetes that we haven’t covered?
- Thank the participant for their time and insights.
